# Jatrorrhizine: A Review of Sources, Pharmacology, Pharmacokinetics and Toxicity

**DOI:** 10.3389/fphar.2021.783127

**Published:** 2022-01-13

**Authors:** Furong Zhong, Yang Chen, Jia Chen, Hailang Liao, Yirou Li, Yuntong Ma

**Affiliations:** ^1^ State Key Laboratory of Characteristic Chinese Medicine Resources in Southwest China, Chengdu University of Traditional Chinese Medicine, Chengdu, China; ^2^ School of Pharmacy, Chengdu University of Traditional Chinese Medicine, Chengdu, China

**Keywords:** jatrorrhizine, natural products, pharmacological properties, toxicology, pharmacokinetics

## Abstract

Jatrorrhizine, an isoquinoline alkaloid, is a bioactive metabolite in common medicinal plants, such as *Berberis vernae* Schneid., *Tinospora sagittata* (Oliv.) Gagnep. and *Coptis chinensis* Franch. These plants have been used for centuries in traditional medicine for their wide-ranging pharmacological properties. This review emphasizes the latest and comprehensive information on the sources, pharmacology, pharmacokinetics and toxicity of jatrorrhizine. Studies on this alkaloid were collected from scientific internet databases, including the Web of Science, PubMed, ScienceDirect, Google Scholar, Elsevier, Springer, Wiley Online Library and Europe PMC and CNKI, using a combination of keywords involving “jatrorrhizine”, “sources”, “pharmacology,” “pharmacokinetics,” and “toxicology”. Jatrorrhizine exhibits anti-diabetic, antimicrobial, antiprotozoal, anticancer, anti-obesity and hypolipidemic properties, along with central nervous system activities and other beneficial activity. Studies of jatrorrhizine have laid the foundation for its application to the treatment of various diseases, but some issues still exist. Further investigations might emphasize 1) specific curative mechanisms of jatrorrhizine and clinical utility, 2) application prospect in the treatment of metabolic disorders, 3) comprehensive investigations of the toxicity mechanisms and 4) interactions of jatrorrhizine with other pharmaceuticals and development of derivatives.

## Introduction

Plants are sources of metabolites with varied biological activities, clinical effectiveness. The therapeutical benefits and safety of plant-derived metabolites have been proven in long-standing traditional medicinal practices across the world (Gurib-Fakim, 2006; Porras et al., 2020). The bioactive metabolites and related derivatives are increasingly used for the production of new drugs and may have broad clinical applications.

**FIGURE 1:**
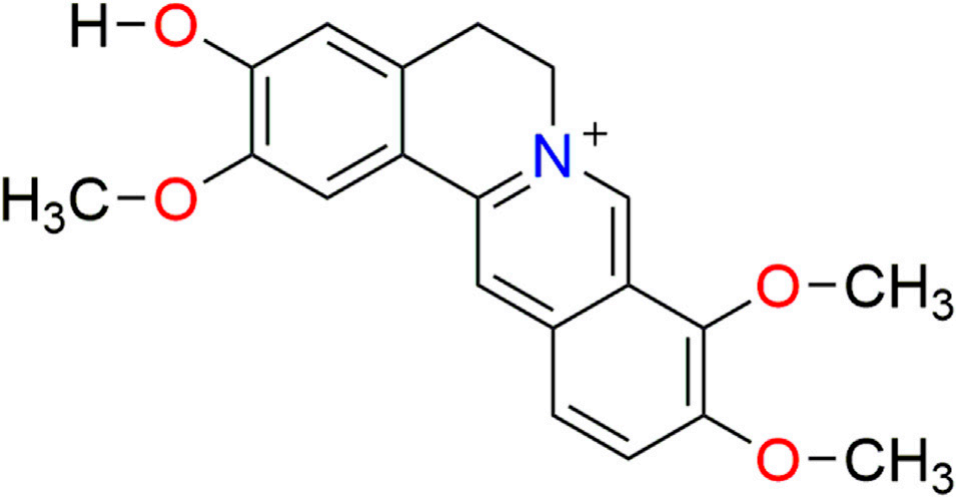
The chemical structure of jatrorrhizine.

Alkaloids are important natural products derived mostly from amino acids. These chemicals display substantial physiological and pharmacological activities. Jatrorrhizine is a well-known isoquinoline alkaloid of the protoberberine type. Its molecular structure is 2,9,10-trimethoxy-5,6-dihydroisoquinolino[2,1-b]isoquinolin-7-ium-3-ol (molecular formula: C_20_H_20_NO_4_, Figure 1). Jatrorrhizine is a major bioactive metabolite with wide distribution across plant families. Several species have been used medicinally for centuries, such as *Berberis vernae* C.K.Schneid. (Li et al., 2020), *Mahonia bealei* (Fortune) Carrière (He and Mu, 2015), *Tinospora sagittata* (Oliv.) Gagnep. (Zhang et al., 2006), *Coptis chinensis* Franch. (Chen et al., 2017) and *Corydalis yanhusuo* (Y.H.Chou and Chun C. Hsu) W.T.Wang ex Z.Y.Su and C.Y.Wu (Xiao et al., 2011). One of the active constituents in herbal formulae, such as Zoujinwan, Jiaotai Pills and San-Huang decoction, is considered to be jatrorrhizine (Yan et al., 2012; Sun et al., 2018; Su et al., 2020). Modern pharmacological studies demonstrate that this alkaloid exhibits anti-diabetic, antimicrobial (Ali et al., 2013), antiprotozoal (Malebo et al., 2013), anticancer (Sun et al., 2019), anti-obesity and hypolipidemic properties (Yang et al., 2016). Central nervous system activities are also reported (Luo et al., 2011; Xiao et al., 2011; Bacq et al., 2012).

Jatrorrhizine has attracted the attention of researchers due to its wide distribution across a variety of plant species and its potential for clinical use certain diseases. We summarise and discuss, in this review, the latest and comprehensive information on the plant sources, synthesis, pharmacological effects, pharmacokinetics and toxicity of jatrorrhizine. This information will be beneficial for future examination of therapeutic potential of this active metabolite and subsequent development of clinical applications.

## Sources

### Plant Sources of Jatrorrhizine

Medicinal plants are major sources of jatrorrhizine. This metabolite is isolated from various plant families, such as Annonaceae, Berberidaceae, Menispermaceae, Papaveraceae, Ranunculaceae and Rutaceae (Table 1). Many species in these families are used in folk medicinal plants and Chinese herbal medicine. Several *Annickia* species from the Annonaceae family, are multi-purpose medicinal plants used widely for the treatment of malaria and other ailments across tropical Africa. Protoberberine alkaloids (including jatrorrhizine and palmatine) are the major anti-protozoal agents in these plants (Malebo et al., 2013; Olivier et al., 2015; Odoh et al., 2018). Numerous species of the *Berberis*, *Mahonia*, *Tinospora*, *Corydalis*, *Coptis*, *Thalictrum* and *Phellodendron* genera are commonly used medicinal plants and important sources of jatrorrhizine (Alamzeb et al., 2015; Bajpai et al., 2016; Du et al., 2018; Feng et al., 2018; Abdykerimova et al., 2020; Sharma et al., 2020). In China, the stems of *Mahonia bealei* (Fortune) Carrière and *Mahonia fortunei* (Lindl.) Fedde (named Mahoniae Caulis), rhizomes of *Coptis chinensis* Franch., *Coptis deltoidea* C.Y.Cheng and P.K.Hsiao and *Coptis teeta* Wall. (named Coptidis Rhizoma) and barks from *Phellodendron amurense* Rupr. (named Phellodendri amurensis Cortex) and *Phellodendron chinense* C.K.Schneid. (named Phellodendri Chinensis Cortex) are known for antipyretic and analgesic properties. These traditional medicines have been widely used to treat abdominal pain and diarrhea, inflammatory disorders and gastrointestinal diseases (Ryuk et al., 2012; He and Mu, 2015; Meng et al., 2018). However, the wild resources of the three *Coptis* species are almost endangered (Chen et al., 2017). Jatrorrhizine is also found in the Rutaceae stem barks of several *Zanthoxylum* species. The anti-cancer activity of these plants might be attributed to quaternary alkaloids (Tian et al., 2017).

**TABLE 1:** The plant sources of jatrorrhizine.

Plant species	Family	Used part	References
*Annickia affinis* (Exell) Versteegh and Sosef	Annonaceae	stem bark	Olivier et al. (2015)
*Annickia chlorantha* (Oliv.) Setten and Maas	Annonaceae	stem bark	Odoh et al. (2018), Olivier et al. (2015)
*Annickia kummeriae* (Engl. and Diels) Setten and Maas	Annonaceae	leaf	Malebo et al. (2013)
*Duguetia trunciflora* Maas and A.H.Gentry	Annonaceae	leaf	Fechine et al. (2002), Pérez and Cassels (2010)
*Xylopia parviflora* Spruce	Annonaceae	bark and root	Nishiyama et al. (2004)
*Berberis aristata* DC.	Berberidaceae	root	Basera et al. (2021)
*Berberis brevissima* Jafri	Berberidaceae	cortex	Ali et al. (2013)
*Berberis dictyophylla* Franch.	Berberidaceae	cortex	Feng et al. (2018)
*Berberis diaphana* Maxim.	Berberidaceae	cortex	Feng et al. (2018)
*Berberis iliensis* Popov	Berberidaceae	root, leaf and fruit	Abdykerimova et al. (2020)
*Berberis jaeschkeana* C.K.Schneid.	Berberidaceae	bark of root	Alamzeb et al. (2015)
*Berberis kansuensis* C.K.Schneid.	Berberidaceae	cortex	Feng et al. (2018)
*Berberis parkeriana* C.K.Schneid.	Berberidaceae	cortex	Ali et al. (2013)
*Berberis vernae* C.K.Schneid.	Berberidaceae	cortex	Feng et al. (2018)
*Mahonia aquifolium* (Pursh) Nutt.	Berberidaceae	root	Slobodníková et al. (2004)
*Mahonia bealei* (Fortune) Carrière	Berberidaceae	root, stem	He and Mu (2015)
*Mahonia fortunei* (Lindl.) Fedde	Berberidaceae	root, root bark stem	He and Mu (2015)
*Mahonia leschenaultia* (Wall. ex Wight and Arn.) Takeda ex Gamble	Berberidaceae	root	Singh et al. (2017)
*Mahonia napaulensis* DC.	Berberidaceae	root	Singh et al. (2017)
*Mahonia oiwakensis* Hayata	Berberidaceae	root	Chao et al. (2009)
*Nandina domestica* Thunb.	Berberidaceae	fruit, ground parts	Iwasa et al. (2008), Peng et al. (2014)
*Burasaia australis* Elliot	Menispermaceae	root	Da-Cunha et al. (2005)
*Burasaia congesta* Decne.	Menispermaceae	root	Da-Cunha et al. (2005)
*Burasaia gracilis* Decne.	Menispermaceae	root	Da-Cunha et al. (2005)
*Dioscoreophyllum cumminsii* (Stapf) Diels	Menispermaceae	stem, leaf, tuber	Furuya et al. (1983)
*Fibraurea recisa* Pierre.	Menispermaceae	stem bark	Su et al. (2007)
*Fibraurea tinctoria* Lour.	Menispermaceae	stem bark	Rao et al. (2009)
*Penianthus zenkeri* (Engl.) Diels	Menispermaceae	leaf, root	Achenbach and Hemrich (1991)
*Sphenocentrum jollyanum* Pierre	Menispermaceae	root	Hussain et al. (1989)
*Stephania cambodica* Gagnep.	Menispermaceae	tuber	Dary et al. (2017)
*Stephania rotunda* Lour.	Menispermaceae	stem, leaf, tuber	Zhang and Rao (2009)
*Stephania yunnanensis* H.S. Lo	Menispermaceae	tuber	Desgrouas et al. (2014)
*Tinospora capillipes* Gagnep.	Menispermaceae	root	Xiang et al. (2016);
*Tinospora cordifolia* (Willd.) Hook.f. and Thomson	Menispermaceae	stem	Bajpai et al. (2016);
*Tinospora sagittata* (oliv.) Gagnep.	Menispermaceae	stem	Yuan et al. (2010)
*Corydalis decumbens* (Thunb.) Pers.	Papaveraceae	rhizome	Mao et al. (2017)
*Corydalis nobilis* (L.) Pers.	Papaveraceae	rhizome	Slavík and Slavíková (1989)
*Corydalis yanhusuo* (Y.H.Chou and Chun C.Hsu) W.T.Wang ex Z.Y.Su and C.Y.Wu	Papaveraceae	tuber	Du et al. (2018)
*Eschscholzia californica* Cham.	Papaveraceae	root	Kukula-Koch (2017)
*Aquilegia Formosa* Fisch.	Ranunculaceae	root	Constantine et al. (1966)
*Coptis chinensis* Franch.	Ranunculaceae	rhizome	He et al. (2014b);
*Coptis deltoidea* C.Y.Cheng and P.K.Hsiao	Ranunculaceae	rhizome	He et al. (2014b)
*Coptis omeiensis* (C.Chen) C.Y.Cheng	Ranunculaceae	rhizome	He et al. (2014b)
*Coptis japonica* (Thunb.) Makino	Ranunculaceae	rhizome	Ikuta et al. (1975)
*Coptis quinquefolia* Miq.	Ranunculaceae	rhizome	Da-Cunha et al. (2005)
*Coptis quinquesecta* W.T.Wang	Ranunculaceae	rhizome	Da-Cunha et al. (2005)
*Coptis teeta* Wall.	Ranunculaceae	rhizome	He et al. (2014b)
*Hydrastis canadensis* L.	Ranunculaceae	root	Le et al. (2014)
*Thalictrum angustifolium* L.	Ranunculaceae	root	Alhowiriny et al. (2002)
*Thalictrum cultratum* Wall.	Ranunculaceae	root	Lou et al. (1987)
*Thalictrum foliolosum* DC.	Ranunculaceae	root	Sharma et al. (2020)
*Thalictrum simplex* L.	Ranunculaceae	root	Qin and Jiang (2011)
*Thalictrum squarrosum* Stephan ex Willd.	Ranunculaceae	root	Qin and Jiang (2011)
*Phellodendron amurense* Rupr.	Rutaceae	stem bark	Ryuk et al. (2012)
*Phellodendron chinense* C.K.Schneid.	Rutaceae	stem bark	Ryuk et al. (2012)
*Zanthoxylum ailanthoides* Siebold and Zucc.	Rutaceae	stem bark	Tian et al. (2017)
*Zanthoxylum chalybeum* Engl.	Rutaceae	stem bark	Tian et al. (2017)
*Zanthoxylum simulans* Hance	Rutaceae	stem bark	Tian et al. (2017)

### Synthesis of Jatrorrhizine

**FIGURE 2:**
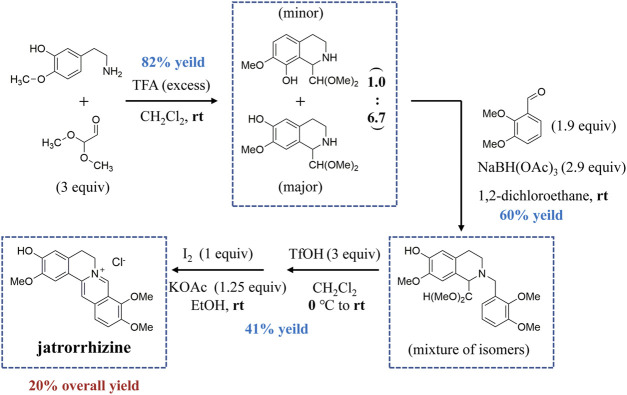
The total syntheses of jatrorrhizine based on a unified strategy.

Access to natural products with complex structures is a major challenge because of slow growth and limited production (Romanowski and Eustáquio, 2020). Chemical synthesis has thus become an effective way to obtain some plant metabolites. Total synthesis of jatrorrhizine has been achieved through an efficient syntheses strategy in four steps (Figure 2). The alkaloid was synthesized from phenethylamine and 2,2-dimethoxyacetaldehyde using the Pictet–Spengler reaction to provide tetrahydroisoquinoline. This intermediate then reductively aminated with 2,3-dimethoxybenzaldehyde to afford the tertiary amine. Friedel–Crafts cyclization and subsequent oxidation deliver isomerically pure jatrorrhizine. This synthesis of jatrorrhizine displayed a 20% overall yield (Mori-Quiroz et al., 2018).

**FIGURE 3:**
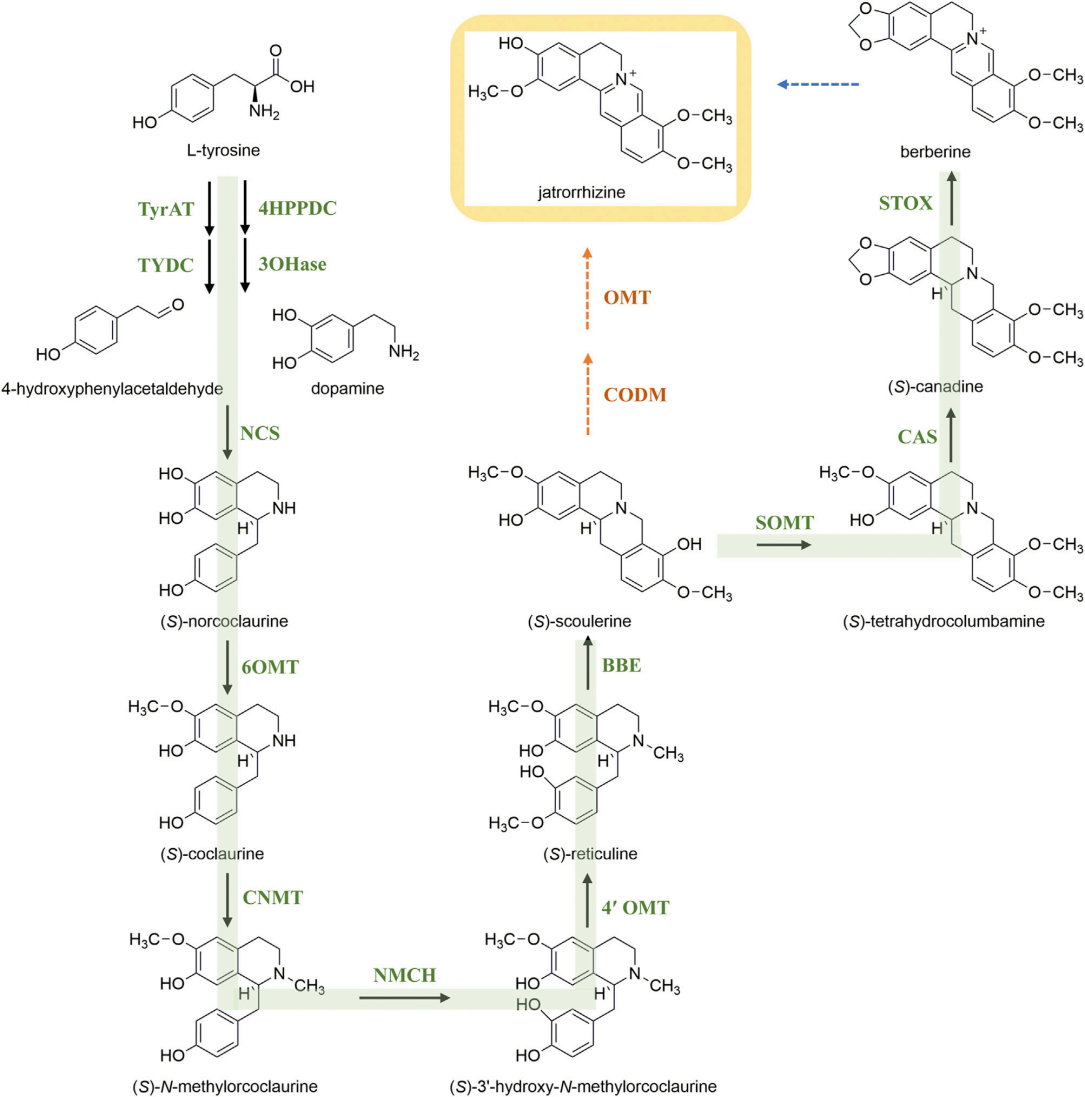
Putative biosynthetic pathway of jatrorrhizine in plants.

Microbial biosynthesis might become a fast and efficient way to obtain natural products. Identification and characterization of the biosynthetic pathway of jatrorrhizine is a prerequisite for its heterologous expression and production. Isoquinoline alkaloids are an important group of specialized plant metabolites. Biosynthesis proceeds by common early steps to form (*S*)-reticuline (Figure 3). This pivotal intermediate is the branch-point intermediate in the biosynthesis of many isoquinoline alkaloids (He et al., 2018). Sequentially, (*S*)-scoulerine is formed from (*S*)-reticuline by berberine bridge enzyme. Pyne et al. (2020) reported a yeast platform for high-level synthesis of tetrahydroisoquinoline alkaloids, and the production of the central intermediate (*S*)-reticuline increased to 4.6 g/L. However, the subsequent pathway leading to production jatrorrhizine remains unknown. Hagel and Facchini proposed that 3-*O*-demethylation of (*S*)-scoulerine combined with 2-*O*- and 9-*O*-methylation might lead to jatrorrhizine (Hagel and Facchini, 2010); enzymes that might catalyse these reactions have not been identified to date. Hence, more research needed to clarify the biosynthetic pathway of jatrorrhizine.

## Pharmacological Activities of Jatrorrhizine

### Anti-Obesity and Hypolipidemic Activity

Obesity is a challenging health problem worldwide. Plants and their active phytochemical constituents are used as natural anti-obesity agents and dietary supplements for weight loss. Jatrorrhizine increased the expression of hepatic low-density lipoprotein receptor (LDLR) in Hep G2 cells *in vitro* and produced a significant reduction in cellular lipid accumulation (Zhou et al., 2014). Jatrorrhizine (46.7 mg/kg×day) was administered to high-fat and high-cholesterol (HFHC)-induced hyperlipidemic hamsters. This treatment reduced the serum total cholesterol (TC) and total triglyceride (TG), decreased the low-density lipoprotein cholesterol (LDL-C) levels, reduced protein levels of 3-hydroxy-3-methyl glutaryl coenzyme A reductase (HMGCR) and significantly increased the expression of cholesterol 7α-hydroxylase (CYP7A1) and LDLR, as well as elevated fecal excretion of cholesterol and TBA (He et al., 2016). In addition, jatrorrhizine decreased body weights of C57BL/6 mice on a HFHC diet and increased HDL-C levels (Yang et al., 2016). The anti-obesity and hypolipidemic effect of jatrorrhizine may thus be related to regulating the expression of LDLR, CYP7A1 and HMGCR, increasing lipid metabolism, and promoting excretion of TBA. All of these effects would lead to increase metabolism and excretion of cholesterol.

Jatrorrhizine ameliorated the pathophysiological changes observed in the livers of hyperlipidemic mice (e.g., swelling of hepatocytes, lipid accumulation, and so on) and caused in a significant decrease in serum aspartate transaminase (AST) and alanine aminotransferase (ALT) levels. Jatrorrhizine also downregulated the hepatic sterol regulatory element binding transcription factor 1c (SREBP-1c) and fatty acid synthase (FAS) levels and upregulated peroxisome proliferator activated receptor-α (PPAR-α) and carnitine palmitoyl transferase 1A (CPT1A) expression. Hence, jatrorrhizine may counter hyperlipidemia through inhibition of fatty acid synthesis and activation of fatty acid *β* oxidation (Yang et al., 2016). Obesity is a complex disorder that significantly increases the risk of multiple metabolic disorders, such cardiovascular disease and diabetes (Karri et al., 2019). The clinical use of jatrorrhizine might helpful in the management of obesity and associated disorders.

### Anti-Diabetic Activity

Type 2 diabetes mellitus (T2DM), an expanding global health problem, is characterized by insulin resistance and impaired insulin secretion (DeFronzo et al., 2015). Some botanical drugs containing jatrorrhizine, such as Coptidis Rhizoma, are widely used in traditional Chinese medicine for treating diabetes (Ma et al., 2016; Meng et al., 2018).

The potential of jatrorrhizine as a hypoglycaemic agent was manifest by inhibition of α-glucosidase and aldose reductase (AR) (Patel MB. and Mishra S., 2012; Patel M. B. and Mishra S. M., 2012). Jatrorrhizine displayed anti-diabetic activity *in vitro* (RINm5F cells and HepG2 cells) and *in vivo* (glucose-loaded rats and hyperlipidemic mice) *via* promoting insulin secretion, improving glucose tolerance and insulin sensitivity and inhibiting hepatic gluconeogenesis, thus improve postprandial hyperglycemia (Patel and Mishra, 2011; Chen et al., 2012; Yang et al., 2016; Li et al., 2020).

Jatrorrhizine protected rats with induced diabetes mellitus and restored vascular endothelial dysfunction through upregulating the Akt/AMPK/eNOS signaling pathway and reducing IL-1β and tumor necrosis factor α (TNF-α) in blood vessels (Wang, et al., 2017). The alkaloid regulated glucose uptake and utilization and reduced insulin resistance through upregulating the expression of insulin receptor substrate 2 (IRS2), phosphoinositide-3-kinase regulatory subunit 1 (PI3KR1), phosphorylated protein kinase B (p-AKT), phospho-AMP-activated protein kinase (p-AMPK) and glucose transporter 4/1/2 (GLUT4/1/2) (Zhu et al., 2018). Jatrorrhizine, a primary active component of Coptidis Rhizoma, displayed potent inhibition of gut microbiota modulation and reduction of blood glucose in d*b*/*db* mice (Lyu et al., 2021).

Jatrorrhizine is thus considered to be an active ingredient with multiple manners that reduce hypoglycaemia (Table 2). However, a comparative study of jatrorrhizine and existing anti-diabetic drugs is not available. Systematic clinical research and molecular studies of jatrorrhizine are still needed to elucidate definite mechanism of action. It is reported that other alkaloids in Coptidis Rhizoma also exhibit anti-diabetic effects, such as berberine, coptisine and palmatine (Lyu et al., 2021). The synergy between this natural metabolite with other alkaloids in Coptidis Rhizoma is of special interest, including interacts with berberine, coptisine and palmatine.

**TABLE 2:** Anti-diabetic, antimicrobial, antiprotozoal, and central nervous system activities and mechanisms of jatrorrhizine in *in vitro* and *in vivo* assays.

Effect	Assay	Cell lines/model	Dosage	Type of biological activity	References
Anti-obesity and hypolipidemic activity					
	*In vitro*	HepG2 cells	15 μM	Increased LDLR expression and decreased cellular lipid accumulation	Zhou et al. (2014)
	*In vivo*	high-fat and high-cholesterol (HFHC)-induced hyperlipidemic hamsters	46.7 mg/kg	Decreased TC, TG, TBA and increased the fecal excretion of cholesterol; upregulation of LDLR, CYP7A1 and HMGCR	He et al. (2016)
	*In vivo*	C57BL/6 mice on a HFHC diet	20 mg/kg; 100 mg/kg	Decreased body weight, TC, TG, LDL-C, AST, ALT and increased HDL-C; amelioration of liver pathophysiological changes (swelling of hepatocytes and lipid accumulation); downregulation of SREBP-1c and FAS; upregulation of PPAR-α and CPT1A	Yang et al. (2016)
Anti-diabetic activity					
	*In vitro*	RINm5F cells	20 μg/ml	Increased insulin secretion	Patel and Mishra (2011)
		Rat hepatocytes	5–80 μg/ml	Inhibition of hepatic gluconeogenesis	
	*In vivo*	Glucose-loaded rats	40 mg/kg	Increased insulin secretion and inhibition of hepatic gluconeogenesis	
	*In vitro*	HepG2 cells	0.6 μM	Glucose-lowering effect	Chen et al. (2012)
	*In vivo*	Diabetes mellitus Wistar rats	50, 100 mg/kg	Reduced IL-1β, TNF-α and upregulation of p-AKT, p-AMPK, eNOS	Wang et al. (2017)
	*In vitro*	IR-3T3-L1 adipocytes	0.5, 1, 5, 10, 20 μmol/L	Amelioration of insulin resistance and upregulation of IRS2, PI3KR1, p-AKT, p-AMPK and GLUT4/1/2	Zhu et al. (2018)
	*In vivo*	Hyperlipidemia model mouse	100 mg/kg	Reduced the body weight and improved glucose tolerance and insulin sensitivity	Yang et al. (2016)
	*In vitro*	α-glucosidase	IC_50_ = 36.25 μg/ml	Inhibitory activity against α-glucosidase	Patel and Mishra (2012b)
	*In vivo*	Wistar rats	20 mg/kg		
	*In vitro*	Lens AR isolated from Wistar rats	IC_50_ = 3.23 mg/ml	Inhibitory activity against aldose reductase	Patel and Mishra (2012a)
Anti-microbial activity					
	*In vitro*	*Candida albicans* SC5314	MIC = 256 μg/ml	Inhibitory activity against *Candida albicans* and *Candida auris*	Liu et al. (2020)
		*Candida auris* 12372	16 μg/ml in *Candida albicans*	Induced cell wall remodeling	
			64 μg/ml in *Candida auris*		
	*In vitro*	*Propionibacterium acnes* coagulase-negative *staphylococci Candida tropicalis*	MIC of 25–50 μg/ml in *Propionibacterium acnes*	Inhibitory activity against *Propionibacterium acnes*, coagulase-negative *staphylococci* and *Candida tropicalis*	Slobodníková et al. (2004)
			MIC of 100–250 μg/ml in coagulase-negative *staphylococci*		
			MIC of 125 μg/ml in *C. tropicalis*		
	*In vitro*	*Staphylococcus aureus* SMRSA 106 and EMRSA 16	200 μg/ml	Inhibition of antibiotic resistant *Staphylococcus aureus*	Ali et al. (2013)
	*In vitro*	*Staphylococcus aureus* (MRSA) SA1199B	MIC = 64 mg/L	Inhibitory activity against methicillin-resistant *Staphylococcus aureus*	Yu et al. (2019)
	*In vivo*	Neutropenic murine thigh infection model	25 or 50 mg/kg of jatrorrhizine and 100 mg/kg of NFX		
	*In vitro*	Neuraminidase of *Clostridium perfringen*s	IC_50_ = 37.0 ± 1.8 μΜ	Inhibitory activity against bacterial NA	Kim et al. (2014)
Anti-protozoal activity					
		*Plasmodium falciparum* K1	IC_50_ = 0.24 ± 0.002 μg/ml	Anti-plasmodial, anti-trypanosomal and anti-leishmanial activity	Malebo et al. (2013)
		*Trypanosoma brucei rhodesiense* STIB 900	IC_50_ = 4.2 ± 0.002 μg/ml		
		*Leishmania donovani* axenic MHOM-ET-67/82	IC_50_ = 20.4 ± 0.03 μg/ml		
Central nervous system activities					
Anti-depression and anxiolytic activity	*In vitro*	Madin-Darby canine kidney cell line	IC_50_ = 2.31 ± 0.21 μM	Inhibition of OCT2	Li et al. (2016)
			IC_50_ = 4.09 ± 1.2 μM	Inhibition of OCT3	
		hOCT2-transfected cells	IC_50_ = 0.120 μM	Decreased 5-HT and NE mediated by OCT2	
			IC_50_ = 0.819 μM		
		hOCT3-transfected cells	IC_50_ = 0.278 μM	Decreased 5-HT and NE mediated by OCT3	
			IC_50_ = 0.184 μM		
		PMAT-transfected cells	IC_50_ = 3.84 μM	Decreased 5-HT and reduce NE uptake mediated by PMAT	
			IC_50_ = 2.99 μM		
	*In vivo*	Male ICR albino mice	5, 10, 20 mg/kg of i.p	Reduced the duration of immobility in mouse tail suspension test	
	*In vitro*	Monoamine oxidase-A	IC_50_ = 57.73 ± 5.26 μM	Inhibitory activity against MAO-A enzyme	Zhang et al. (2019)
	*In vitro*	MAO-A from rat brain mitochondria	IC_50_ = 4 μM		Kong et al. (2001)
Anti-Alzheimer’s disease	*In vitro*	Acetylcholinesterase	IC_50_ = 0.57 μM	Inhibitory activity against AChE	Lin et al. (2020)
	*In vitro*	Recombinant human IDO-1	IC_50_ = 206 μM	Inhibitory activity against IDO-1	Yu et al. (2010)
		HEK 293-hIDO1 cells	IC_50_ = 17.8 μM		
	*In vitro*	HT22 cells	5, 10 μmol/L	Antioxidation and inhibition of the mitogen-activated protein kinases (MAPK) pathways	Jiang et al. (2015)
		SH-SY5Y cells induced by Aβ 25-35	10 mM	Upregulation of miR-223-3p, inhibition of the HDAC4 expression, suppression of apoptosis and OS, and improved cell proliferation	Duan and Chen (2021)
	*In vivo*	APP/PS1 transgenic mice	5, 10 mg/kg	Decreased the levels of Aβ plaques in the cortex and hippocampus, alleviated the learning and memory deficits	Wang et al. (2019b)
	*In vivo*	C57BL/6 wild-type (WT) mice	High dose	Regulated the abundance of the microbiota and increased the amounts of beneficial bacteria	
Neuroprotective effect	*In vitro*	H_2_O_2_-induced rat pheochromocytoma line PC12 injury	0.01–10.0 μM	Increased cell viability and activities of SOD, HO-1; decreased LDH, MDA and ROS; inhibited apoptosis by inhibiting caspase-3 activation	Luo et al. (2011)
Treatment of ischaemic stroke	*In vitro*	mouse brain endothelial cells	5, 10, 20 μM	Reduced t-BHP-induced apoptosis; decreased ROS, MDA and 4-HNE; improved MMP and eNOS; inhibit IL-1β, TNF-α and IL-6; prevented decreases in PPAR-γ	Wu et al. (2020)
Anti-parkinsonian	*In vitro*	MAO-B from rat brain mitochondria	IC_50_ = 62 μM	Inhibitory activity against MAO-B enzyme	Kong et al. (2001)
Effects on bones					
	*In vivo*	Titanium Particle-induced murine calvarial osteolytic model (C57BL/6 mice)	100 mg/kg	Increased BMD and BV/TV, reduced bone erosion and the number of osteoclasts	Li et al. (2018)
	*In vitro*	bone marrow-derived macrophages	5–20 µM	Inhibited RANKL-induced osteoclast formation and bone resorption by the suppression of MAPKs signaling pathways and downregulation of NFATc1, TRAP, CTR and CTSK	
	*In vivo*	collagen-induced arthritis (CIA) rats	20 mg/kg; 50 mg/kg	Inhibited NF-κB and MAPKs stimulated by TNF-α and inhibited bone destruction	Qiu et al. (2018)
Other pharmacological activities					
Effect on gastrointestinal tracts	*In vitro*	Gastrointestinal tract smooth muscles isolated from rat	100 μM	Increased the amplitude of contractile responses of jejunum and ileum longitudinal muscles, antrum circular muscles and smooth muscles in distal colon, and activated acetylcholine receptors	Yuan et al. (2011)
	*In vivo*	Male Wistar rats	0.1, 0.3 and 1 mg/kg	Offset of postoperative ileus-induced delayed gastric emptying and intestinal transit	Zhang et al. (2012)
Hepatoprotective activity	*In vitro*	t-BHP-injured rat hepatocyte BRL-3A cells	EC_50_ = 15.7 ± 3.3 μM	Decreased the release of LDH	Wang et al. (2016)

**TABLE 3:** The anti-cancer effects of jatrorrhizine and its complexes.

Cancer type	Cells or tumor models	Application	Dosage	Suppressive effect	Mechanisms	References
Melanoma	C8161 human metastatic melanoma cell line	*In vitro*	80, 160, 320 μmol/L, 48 h	Inhibition of cell proliferation and neovascularization	Cell cycle arrest, and upregulation of p21 and p27, p53	Liu et al. (2013)
	Matrigel plug assay in BALB/C nude mice	*In vivo*	50 μg, 14 days		Reduced numbers of blood vessels	
Colorectal cancer	SW480 human colon cancer cell line	*In vitro*	25–200 μg/ml, 24 and 48 h	Inhibition of cell proliferation and cell viability		Singh et al. (2016)
	SW620 colorectal cancer cell line	*In vitro*	100 μM	Inhibition of cell proliferation	Formation of complexes with oncogene *KRAS* promoter NHE G-quadruplex	Wen and Xie (2017)
	Human colorectal carcinoma cell lines HCT-116 and HT-29	*In vitro*	IC_50_ of HCT-116: 6.99 ± 0.29 μM, 72 h	Suppression of cell growth and proliferation, inhibit migration and invasion	Promotion of apoptosis, induced nuclear morphological changes, block of cell cycle in S phase, repressed ∆Ψm, reduced β-catenin, F-actin and N-cadherin, and increased GSK-3β and E-cadherin	Wang et al. (2019a)
			IC_50_ of HT-29: 5.46 ± 0.13 μM, 72 h			
			5, 10, 15 μM			
			24, 48, and 72 h			
	HCT-116 nude mice xenograft model	*In vivo*	5 mg/kg, 4 weeks	Inhibition of tumor growth and metastasis	Reduced tumor volume and weight, upregulation of GSK-3β and E-cadherin, and downregulation of β-catenin, F-actin and N-cadherin	
Liver cancer	HepG2 and HCCLM3 liver cancer cells	*In vitro*	0.5–16.0 µM, 48 h	Inhibition of cell viability, proliferation, invasion and migration	Promotion of apoptosis, downregulation of miR-221-3p and miR-15b-5p expression, and upregulation of Axin2 protein	Deng and Wan (2021)
Breast cancer	MDA-MB-231 triple-negative breast cancer cell line, MCF-7 estrogen receptor positive breast carcinoma cell line, and 4T1 mouse mammarycarcinoma cells	*In vitro*	10, 20, 30 μM	Inhibition of cell proliferation	Repressed ∆Ψm, suppressed Wnt/β-catenin signaling and EMT expression *via* targeted TNIK, upregulation of GSK-3β and E-cadherin, and downregulation of β-catenin, F-actin and N-cadherin, up-regulate Bax, downregulation of Bcl-2, decreased Procaspase-3, Procaspase-8, Procaspase-9 and PARP	Sun et al. (2019)
			24 and 48 h			
	Orthotopic 4T1 tumour bearing mouse	*In vivo*	2.5 mg/kg b.w	Inhibition of the growth and metastasis	Reduced tumor growth rate and improve survival rate, upregulation of GSK-3β and E-cadherin, downregulation of TNIK, p-TNIK, F-actin, β-catenin, and N-cadherin	
			5 mg/kg b.w			
			4 weeks			
Thyroid cancer	SW1736, BHP7-13, and 8305C cell lines	*In vitro*	1.5, 3, 6, 12, 24, 48 μM, 48 h	Inhibition of cell proliferation	Cell cycle arrest, increased accumulation of ROS, promoted the levels of cleaved caspase-3 and p-H2AX, suppressed pS6, p-ERK1/2, p-4E-BP1, p-AKT, KU70, ERCC1, RAD51 and KU80, downregulation of the PI3K/AKT/mTOR signaling pathway and promotion of DNA damage	Lu et al. (2020)
	Female athymic nude mice	*In vivo*	24.0 mg/kg, 14 days	Inhibition of tumor growth	Increased pH2AX and acetylated histone H3, histone H4 and cleaved caspase-3	
HeLa cancer	Human cervical (HeLa) cell line	*In vitro*	Pt1: IC_50_ = 15.01 ± 1.05 nM	Inhibition of cell proliferation	Targeting p53 and telomerase, repressed telomerase related-proteins (c-myc and hTERT), promoted DNA damage (activation of 53BP1, H2A.X, TRF1, and TRF2), decreased ∆Ψm, sub-G1 phase arrest and cell apoptosis	Qin et al. (2019b)
			Pt2: 1.00 ± 0.17 nM			
	Human cervical (HeLa)-xenograft model	*In vivo*	Pt2: 2.0 mg/kg per 2 days, 21 days	Inhibition of tumor growth		
Bladder cancer	Human bladder T-24 tumor cell	*In vitro*	Pt1:100.0 nM, 6 h	Inhibition of cell proliferation	Induced TRF1- and TRF2-telomeres damage, decreased hTERT and c-myc levels, increased ROS, cytochrome c, caspase-9, caspase-3, Apaf-1, inhibited Bcl-2, and cell cycle arrest (suppression of cyclin D1 and CDK2)	Qin et al. (2019a)
			Pt2: 10.0 nM, 6 h			
	T-24 xenograft mouse models (nude mice)	*In vivo*	Pt1: 2.0 mg/kg per 2 days	Inhibition of tumor growth		
			Pt2: 2.0 mg/kg per 2 days			

### Anti-Microbial and Anti-protozoal Activity

Jatrorrhizine, in plants such as *Mahonia aquifolium* (Pursh) Nutt., *Berberis brevissima* Jafri and *Coptis chinensis* Franch. (Slobodníková et al., 2004; Ali et al., 2013; Tseng et al., 2021), is a notable among natural products for its varied anti-microbial properties. This metabolite strongly inhibited the growth of some bacteria, such as *Candida albicans* SC5314 (MIC = 256 μg/ml), *Candida auris* 12372 (MIC = 256 μg/ml), *Candida tropicalis* (MIC = 125 μg/ml), *Propionibacterium acnes* (MIC between 25 and 50 μg/ml), coagulase-negative staphylococci (MIC between 100 and 250 μg/ml) and *Staphylococcus aureus* (200 μg/ml) (Slobodníková et al., 2004; Ali et al., 2013; Liu et al., 2020). This alkaloid induced cell wall remodeling at 16 μg/ml in *Candida albicans* and 64 μg/ml in *Candida auris* (Liu et al., 2020). The mechanism underlying the antimycotic effect was inhibition of drug efflux and expression of the NorA multi-drug efflux pump (Yu et al., 2019). Further, a combination of jatrorrhizine (25 or 50 mg/kg) and norfloxacin (NFX, 100 mg/kg) significantly decreased bacterial count in a murine thigh infection model, suggesting *in vivo* synergistic bactericidal activity. Moreover, the combination of five berberine alkaloids (berberine: coptisine: jatrorrhizine: palmatine: epiberberine = 0.702 : 0.863: 1: 0.491: 0.526) exhibited broad-spectrum antibacterial activity, and this activity was verified *in vivo* using cyclophosphamide-immunocompromised mouse model and *in vitro* against *Escherichia coli*, *Staphylococcus aureus*, *Staphylococcus dysenteriae*, and *Staphylococcus pneumonia*. Hence, jatrorrhizine may act synergistically with other alkaloids (Luo et al., 2013). Moreover, jatrorrhizine showed a synergistic effect with colistin antibacterial activity against carbapenem-resistant *Klebsiella pneumoniae*, exhibiting one-to two-fold reductions of colistin MIC (Tseng et al., 2021).

Neuraminidase (NA) is a novel target for the development of therapeutic agents to treat bacterial or viral infections (Kim et al., 2014; Luo et al., 2020). As documented in literature, jatrorrhizine showed inhibitory activity on bacterial NA with an IC_50_ value of 37.0 ± 1.8 μΜ and suppressed viral NA activity against rvH1N1 and H5N1 with IC_50_ values of 66.2 ± 4.2 μΜ and 76.3 ± 2.1 μΜ (Kim et al., 2014). Molecular modelling and docking studies indicated that jatrorrhizine might be a potent agent against transmembrane protease serine 2 (TMPRSS2) enzyme for treating SARS-CoV-2 (Pooja et al., 2021). It bound to human immunodeficiency virus-1 (HIV-1) as an effective inhibitor of HIV (Namthabad and Mamidala, 2014). Therefore, jatrorrhizine is a promising therapeutic agent and a natural metabolite of the combination therapy for microbial diseases.

*Annickia affinis* (Exell) Versteegh and Sosef and *Annickia chlorantha* (Oliv.) Setten and Maas containing jatrorrhizine are used for the treatment of malaria across tropical Africa (Olivier et al., 2015; Odoh et al., 2018). *In vitro* antiprotozoal studies on jatrorrhizine have shown its anti-plasmodial activity against multi-drug resistant strains of *Plasmodium falciparum* K1 (IC_50_ = 0.24 ± 0.002 μg/ml), anti-trypanosomal activity against the *Trypanosoma brucei* rhodesiense STIB 900 (IC_50_ = 4.2 ± 0.002 μg/ml) and anti-leishmanial activity against *Leishmania donovani* axenic MHOM-ET-67/82 strain (IC_50_ = 20.4 ± 0.03 μg/ml) (Malebo et al., 2013).

*In vivo* validation of naturally occurring anti-microbials and the development of effective alternatives to anti-biotics is crucial in the current era of microorganism resistance. Several studies report anti-microbial and anti-protozoal activity of jatrorrhizine *in vitro*, but clinical efficacy, therapeutic doses, safety and mechanisms remain largely unknown.

### Effects on the Central Nervous System

#### Anti-depressant Activity

Jatrorrhizine demonstrated anti-depressant activity *via* several targets in anti-depressant therapeutics. It showed strong inhibitory activity against monoamine oxidase A (MAO-A) (IC_50_ = 4 μM). This inhibitory activity was greater than that of berberine (IC_50_ = 126 μM), which lacks the phenolic hydroxyl of jatrorrhizine (Kong et al., 2001; Zhang et al., 2019). Furthermore, several studies have shown that organic cation transporters (OCTs) play roles in anti-anxiety and anti-depressant processes (Bacq et al., 2012) and plasma membrane monoamine transporter (PMAT) is a novel anti-depressant target. Jatrorrhizine was proved to be a high-affinity substrate for OCTs and a potent inhibitor of OCT2 (IC_50_ = 2.31 ± 0.21 μM) and OCT3 (IC_50_ = 4.09 ± 1.2 μM) (Li et al., 2016). Moreover, jatrorrhizine strongly reduced serotonin (5-HT) and norepinephrine (NE) uptake mediated by hOCT2, hOCT3, and hPMAT *in vitro*. Meanwhile, jatrorrhizine reduced 5-HT and NE uptake at 50 μM in mouse synaptosomes, and reduced the duration of immobility and reversed the effect of stress in tail suspension tests, consistent with an anti-depressant effect. However, more *in vivo* experiments are needed to verify and clarify the complex anti-depressant activity of jatrorrhizine.

#### Anti-Alzheimer’s Disease

The Alzheimer’s disease (AD) is currently attributed to extracellular aggregates of amyloid β (Aβ) plaques and intracellular neurofibrillary tangles in cortical and limbic areas of the human brain (Tiwari et al., 2019). Defects in acetylcholine and cholinergic neurotransmission can be observed along with the accumulation of β-amyloid. The use of acetylcholinesterase (AChE) inhibitors, which activate central cholinergic function, is a treatment strategy for AD. Jatrorrhizine demonstrated inhibitory activity against AChE with IC_50_ values of 0.57 μM (Lin et al., 2020), 106.1 μM (Zhao et al., 2016) and 2.08 μM (Xiao et al., 2011), respectively. The differences in these values might be explained by the different sources and concentrations of the enzyme and substrate used for testing. Further, a jatrorrhizine derivative with -NH_2_ linked at the 3-position (IC_50_ = 0.301 μM) exhibited the greater AChE inhibitory activity than jatrorrhizine (IC_50_ = 0.872 μM) (Jiang et al., 2017). Hence, structural modification of jatrorrhizine may be effective for modulating its activity.

Indoleamine 2, 3-dioxygenase 1 (IDO-1) is a rate-limiting enzyme in the kynurenine pathway of tryptophan metabolism. The accumulation of a downstream neurotoxic metabolite *via* overexpression or over activation of IDO1 is involved in neurodegenerative disease (Zhang et al., 2017; Röhrig et al., 2019). Jatrorrhizine was able to irreversibly inhibit IDO1, and had IC_50_ values of 206 μM (recombinant human IDO-1) and 17.8 μM (in HEK 293-hIDO1 cells) (Yu et al., 2010).

*In vivo* treatment of APP/PS1 transgenic mice with 5 mg/kg or 10 mg/kg jatrorrhizine reduced levels of Aβ plaques in the cortex and hippocampus, and alleviated the learning and memory deficits (Wang S. et al., 2019). Learning and memory impairment in AD is related to dysfunction in gut microbiota (Vogt et al., 2017). Microbial colonies of APP/PS1 mice showed altered composition compared to C57BL/6 wild-type (WT) mice. High dose jatrorrhizine treatment modulated microbiota populations and enriched the numbers of beneficial bacteria, such as *Faecalibaculum*, *Lactobacillus acidophilus* and *Bifidobacterium* (Wang S. et al., 2019). Thus, jatrorrhizine might affect the learning and memory capabilities by regulating the intestinal flora.

Neuroprotective effects of jatrorrhizine are mainly attributed to its anti-oxidant property and anti-apoptosis activity. The alkaloid alleviates alleviated oxidative damage and suppresses neuronal apoptosis. Jatrorrhizine exhibited neuroprotective activity on okadaic acid (OA)-induced cytotoxicity and apoptosis in HT22 cells. This effect ascribed to increase cell viability, enhance anti-oxidant status (SOD and GSH) and maintenance of mitochondrial membrane potential (MMP). Reduced lactate dehydrogenase (LDH) release, lipid peroxidation (MDA) levels and reactive oxygen species (ROS) were also observed. Other responses included downregulation of expression of phosphorylated extracellular signal-regulated kinases 1/2 (p-ERK1/2), phosphorylated c-Jun N-terminal kinases (p-JNK) and phosphorylated p38 (p-p38), along with upregulation of B cell lymphoma 2 (Bcl-2), reduction in cleaved caspase-3 and BCL-2-associated X protein (Bax) levels, and inhibition of NF-κB p65 subunit activation (Jiang et al., 2015). A possible mechanism was inhibition of mitogen-activated protein kinase (MAPK) pathways. Similarly, jatrorrhizine was effective in mitigating hydrogen peroxide (H_2_O_2_)-induced rat pheochromocytoma PC12 injury *via* reducing oxidative stress and inhibiting apoptosis (Luo et al., 2011). Furthermore, treatment with jatrorrhizine (10 mM) alleviated Aβ 25-35-induced nerve cell injury through upregulating miR-223-3p and inhibiting histone deacetylase 4 (HDAC4) expression. The alkaloid also suppressed apoptosis and oxidative stress (OS) and improved SH-SY5Y cell proliferation (Duan and Chen, 2021).

#### Other Effects on the Central Nervous System

Jatrorrhizine might have therapeutic potential for ischaemic stroke associated with endothelial dysfunction. The alkaloid produced protective effect in mouse brain endothelial cells (MBECs) treated with tert-butyl hydroperoxide (t-BHP) *via* reducing cell apoptosis, inhibiting oxidative damage and ameliorating mitochondrial dysfunction. Jatrorrhizine also prevented the expression of IL-1β, TNF-α and IL-6, and upregulated endothelial nitric oxide synthase (eNOS) and prevented decreases in PPAR-γ protein expression in MBECs (Wu et al., 2020). Jatrorrhizine also non-competitively inhibited MAO-B from rat brain mitochondria with an IC_50_ value of 62 mM (Kong et al., 2001). This activity was intended to be helpful for the prevention and adjunct treatment of Parkinson’ disease.

### Anti-Cancer Activity

**FIGURE 4:**
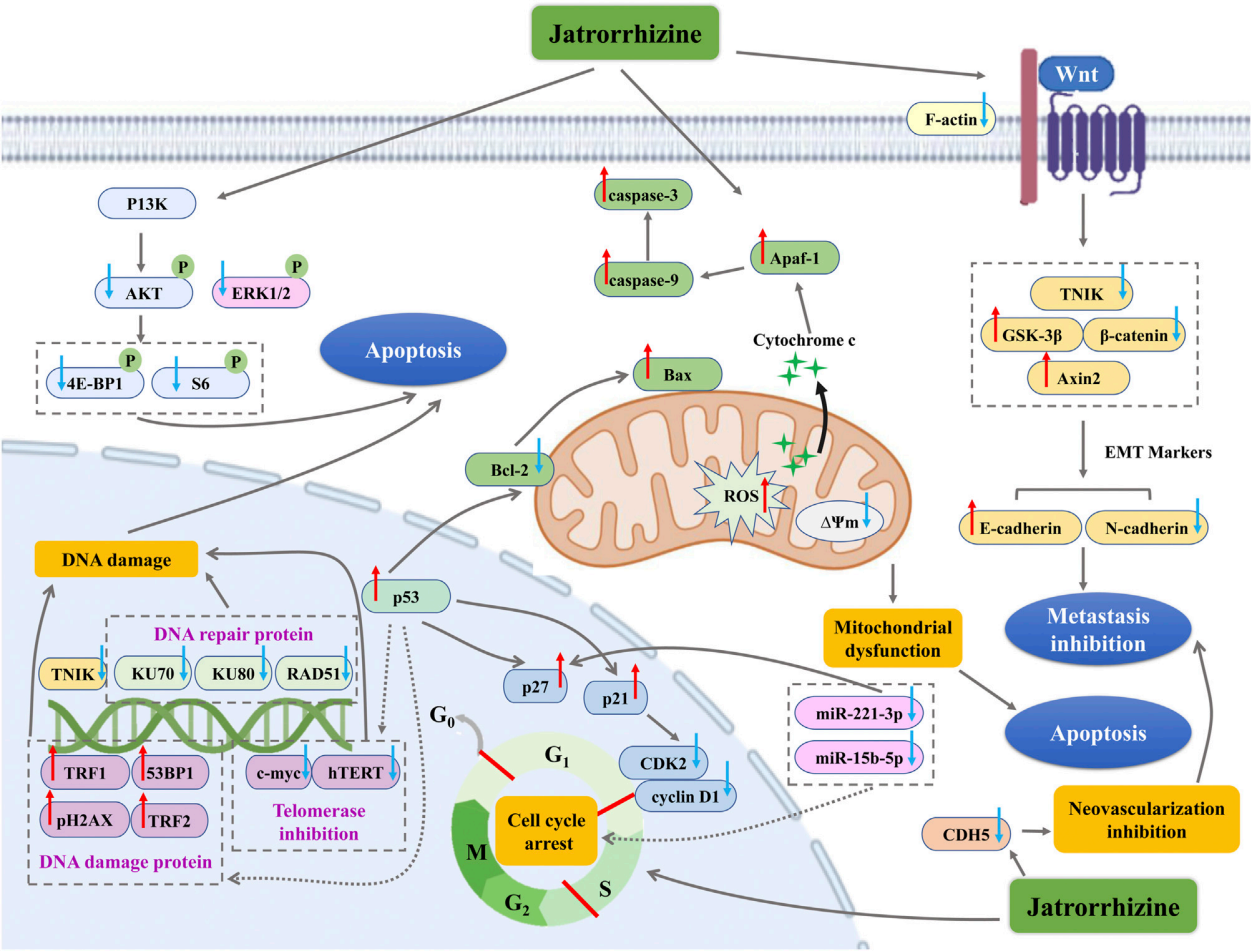
The antitumor mechanism of jatrorrhizine. **↑** with red color indicate increase/promotion, ↓with blue color indicate inhibition/reduction.

Globally, cancer is one of the major diseases that cause a large number of deaths, and the incidence of cancer is increasing in recent years (Sharma et al., 2019). Inhibition of apoptosis, unlimited proliferation of cancer cells, invasion of normal organs and destruction of normal tissues are the main reasons that cancer threatens human health (Rosell and Karachaliou, 2015; Roy and Saikia, 2016). Over the past decades, development of anti-tumor agents from natural products has been one of the fresh approaches for therapeutic candidate discovery. Jatrorrhizine exhibited anti-cancer activity in various cancer cells (*in vitro*) and a few *in vivo* models (Table 3). Its multidirectional mechanisms involve in inhibiting cancer cell proliferation and tumor growth, preventing metastasis, while the important mechanism is promoting apoptosis of cancer cells (Figure 4).

#### Inhibition of Cell Proliferation

The rapid and unlimited proliferation of cancer cells is attributed to the loss or gene mutations of critical checkpoint controlling cycling of cell phase (Andrade-Tomaz et al., 2020). As an important process in cancer development, cell cycle modulation is a well-established therapeutic schedule. Jatrorrhizine (5–15 μM) affected human colorectal carcinoma HCT-116 and HT-29 cells proliferation by blocking cell cycle in S phase and it (5 mg/kg) could prolong the survival of nude mice xenografted HCT-116 cells (Wang P. et al., 2019). In C8161 human metastatic melanoma cells, jatrorrhizine (160 mmol/L) inhibited cell proliferation through inducing cell cycle arrest in the G0/G1 phases and upregulating expression of cyclin-dependent kinase (CDK) inhibitors (*p21*and *p27*) and the tumor suppressor *p53* (Liu et al., 2013). The derivative, Pt(II) complexes with jatrorrhizine blocked cell cycle at G1 phase in human bladder T-24 tumor cells, which was associated with inhibiting the levels of cyclin D1 and CDK2 (Qin et al., 2019a). microRNAs play a prominent role in modulation of cell proliferation by directly targeting cell cycle regulators, such as cyclin, c-myc, p27 and p57 (Yu et al., 2010). Jatrorrhizine (16.0 µM) inhibited cell viabilities of HepG2 and HCCLM3 liver cancer cells by down-regulating miR-221-3p and miR-15b-5p expressions (Deng and Wan, 2021).

#### Inhibition of Cancer Cell Metastasis

Abnormal vascularization and epithelial-mesenchymal transition (EMT) are essential for metastatic spread of cancer cells (Dewaguet et al., 2021). In BALB/C nude mice xenografted metastatic melanoma C8161 cell, jatrorrhizine (50 μg) reduced neovascularization of tumor, probably due to its suppression of CDH5 expression, which encodes the vascular endothelial cadherin (Liu et al., 2013). Traf2 and Nck interacting serine protein kinase (TNIK) has been considered as an important activator of Wnt signaling pathway to promote tumor progression and invasion (Yang et al., 2021). MDA-MB-231 human breast cancer cells targeted knockout of TNIK validated that the disruption of TNIK restrained the key proteins expression of Wnt/β-catenin signalling and EMT (Sun et al., 2019). Interestingly, jatrorrhizine exhibited good binding affinity and interaction with TNIK. The alkaloid effectively downregulated TNIK, p-TNIK, β-catenin, F-actin and N-cadherin expression levels, and upregulated GSK-3β and E-cadherin in *in vitro* (MDA-MB-231 cells and MCF-7 cells) and *in vivo* models (Orthotopic 4T1 tumour bearing mouse) (Sun et al., 2019). Moreover, jatrorrhizine (5 mg/kg) also reduced tumor volume and weight, and inhibited lung metastasis in nude mice xenografted HCT-116 colorectal carcinoma cells *via* suppressing Wnt signaling pathway and the process of EMT (Wang P. et al., 2019). Hence, jatrorrhizine is expected to be an anticancer drug targeting TNIK and EMT.

#### Promotion of Apoptosis

Apoptosis is considered as a major barrier for the development and progression of cancer (Boudreau et al., 2019). Jatrorrhizine (10–30 μM) triggered mitochondrial dysfunction and apoptosis in MDA-MB-231 breast cancer cells. The relevant mechanism was related to disruption of ∆Ψm, upregulation of the pro-apoptotic protein Bax, and downregulation of the anti-apoptotic protein Bcl-2, as well as decrease of apoptosis-related proteins including Procaspase-3, Procaspase-8, Procas-pase-9 and PARP (Sun et al., 2019). Similarly, jatrorrhizine showed disruptive effect on ∆Ψm and nuclear morphological changes in human colorectal carcinoma HCT-116 and HT-29 cells, indicating mitochondrial dysfunction and early apoptosis (Wang P. et al., 2019). Besides, jatrorrhizine-Platinum(II) complex promoted DNA damage of thyroid cancer SW1736 and BHP7-13 cells *via* increasing pH2AX protein (DNA damage protein) and decreasing DNA repair protein KU70, KU80 and RAD51, while activated apoptosis by upregulating ROS and cleaved caspase-3, and downregulating PI3K/AKT/mTOR pathway (pS6, p-ERK1/2, p-4E-BP1, and p-AKT levels) (Lu et al., 2020). In mice bearing SW1736 tumor xenografts, this derivate suppressed tumor growth and tumor tissues expression of pH2AX, which confirmed its anti-cancer activity *in vivo* (Lu et al., 2020). The complexes of jatrorrhizine with platinum (Pt1 and Pt2) induced apoptosis in HeLa cancer cells, which was manifested in that these derivates targeted p53 and telomerase and further caused DNA damage *via* suppression of c-myc and human telomerase reverse transcriptase (hTERT), and activation of 53BP1, pH2AX, TRF1, and TRF2 (Qin et al., 2019b). Consistent with that in HeLa cancer cells, a novel Pt(II) complex as well modulated telomerase related-proteins and DNA damage. It also successfully achieved the induction of apoptosis to decrease ∆Ψm and Bcl-2, increase the release of ROS and cytochrome c, and up-regulate caspase-9, caspase-3 and apoptotic protease activating factor 1 (Apaf-1) (Qin et al., 2019a).

Several studies have illustrated that jatrorrhizine and its derivates exert anti-cancer effect with low systemic toxicity in *in vivo* models, which inhibited tumor growth and metastasis and prolong the survival time for mice bearing tumor xenografts (Liu et al., 2013; Qin et al., 2019a; Wang P. et al., 2019; Sun et al., 2019). Additionally, the complexes of jatrorrhizine with platinum had good effects in the induction of cisplatin-resistant cancer SK-OV-3 cells and could reduce the side effects of anti-tumor drugs such as cisplatin (Qin et al., 2019a; Lu et al., 2020).

On the whole, jatrorrhizine and its derivatives may be a logical agent for tumor therapy. However, in the retrieved studies, the experiments also lacked the information on the selectivity index. More positive controls (anti-cancer drugs for clinical use) are required to further confirm the anticancer effects of jatrorrhizine. We observed that jatrorrhizine inhibited different types of cancer, but *in vivo* models of different cancer stages including tumorigenesis, development and metastasis were not fully considered. More importantly, elaborative consideration needs to be taken into the validation criteria of the models used. In addition, its clinical efficacy, specific targets and long-term drug safety during cancer treatment are critical issues to be addressed.

### Effects on Bones

Jatrorrhizine inhibited osteolysis in titanium particle-induced murine calvarial osteolytic (C57BL/6 mice). Treatment with the alkaloid (100 mg/kg) significantly increased bone mineral density (BMD) as well as bone volume/tissue volume (BV/TV), and reduced bone erosion and the number of osteoclasts (Li et al., 2018). In bone marrow-derived macrophages, jatrorrhizine inhibited receptor activator of nuclear factor κ-B ligand (RANKL)-induced osteoclast formation and bone resorption. Mechanism analysis revealed that these effects were mediated *via* suppression of MAPK (p38 and ERK) signaling and downregulation of nuclear factor of activated T-cells cytoplasmic 1 (NFATc1) and NFATc1-associated osteoclastic genes including tartrate-resistant acid phosphatase (*TRAP*), calcitonin receptor (*CTR*) and cathepsin K (*CTSK*) (Li et al., 2018). Also, jatrorrhizine suppressed the activation of NF-κB and MAPK stimulated by TNF-α, thereby inhibiting inflammatory responses and bone destruction in collagen-induced arthritis (CIA) rats (Qiu et al., 2018). Bone protection and anti-inflammatory effects suggest that jatrorrhizine may be beneficial in reducing infection after orthopaedic titanium implantation. Overall, this natural product may be useful agents for treatment of bone disorders.

### Other Pharmacological Activities of Jatrorrhizine

#### Effects on Gastrointestinal Tracts

Jatrorrhizine at concentrations from 1.0 to 300 μM increased the amplitude of spontaneous contractions of gastrointestinal tract smooth muscles isolated from rats in a concentration-dependent manner. Jatrorrhizine (100 μM) markedly increased contractile responses of jejunum and ileum longitudinal muscles, antrum circular muscles and smooth muscles in the distal colon. These effects were mediated by activation of acetylcholine receptors (probably M3 receptors) and associated with calcium agonistic effects, including enhancing Ca^2+^ influx through L-type Ca^2+^ channel and Ca^2+^ release *via* IP3 and ryanodine pathways (Yuan et al., 2011). Moreover, *in vivo* experiments in rats demonstrated that jatrorrhizine (0.1, 0.3 and 1 mg/kg) offset postoperative ileus-induced delayed gastric emptying and intestinal transit in a dose-dependent manner (Zhang et al., 2012). Hence, jatrorrhizine may be useful for treatment of functional disorders of the gastrointestinal tract.

#### Hepatoprotective Activity

Jatrorrhizine is one of the constituents in three traditional Chinese medicine formulae with hepatoprotective activity for treating jaundice, namely Zhi-Zi-Da-Huang-Tang, Yin-Chen-Hao-Tang and Da-Huang-Xiao-Shi-Tang. The alkaloid decreased the release of LDH (EC_50_ = 15.7 ± 3.3 μM) in a study on t-BHP-injured rat hepatocyte BRL-3A cells. LDH release is an indicator of liver damage and reduced release is evidence of a hepatoprotective effect against oxidative damage (Wang et al., 2016).

## Pharmacokinetics of Jatrorrhizine

Pharmacokinetic evaluation of drugs provides increasingly important information for clinical research. The pharmacokinetic profile of jatrorrhizine after oral or intravenous administration was assessed in rats and rabbits using liquid chromatography-tandem mass spectrometry (LC-MS/MS), LC-MS/MS combined with brain micro-dialysis, ultra-high performance liquid chromatography-mass spectrometry (UPLC-MS/MS), UPLC-orbitrap mass spectrometry and liquid chromatography quadrupole time-of-flight mass spectrometry (LC-qTOF-MS) (Deng et al., 2008; Liu et al., 2011; Shi et al., 2012; He W. et al., 2014; Zhang et al., 2018). The pharmacokinetic parameters of these studies are shown in Table 4.

**TABLE 4:** Pharmacokinetic parameters of jatrorrhizine.

Route of administration	Inclusion of drug components	Species	Dose	Pharmacokinetic parameters	References
Oral	Jiaotai Pills extracts	Rat (brain)	300 mg/kg Rhizoma Coptidis extracts and 4.7 mg/kg cinnamon oil (equivalent to 15.52 mg/kg dose of jatrorrhizine)	T_max_ = 2.17 ± 1.11 min	Zhang et al. (2018)
				T_1/2_ = 2.89 ± 1.76 h	
				AUC_0-t_ = 16.96 ± 1.57 ng h^−1^·mL^−1^	
				AUC_0-∞_ = 24.45 ± 1.73 ng h^−1^·mL^−1^	
				K_e_ = 0.98 ± 1.79 h^−1^	
				C_max_ = 5.56 ± 2.40 ng/ml	
		Insomnic rat (brain)		T_max_ = 2.13 ± 1.03 min	
				T_1/2_ = 6.35 ± 2.25 h	
				AUC_0-t_ = 34.26 ± 7.03 ng h^−1^·mL^−1^	
				AUC_0-∞_ = 43.53 ± 6.13 ng h^−1^·mL^−1^	
				K_e_ = 0.21 ± 0.16 h^−1^	
				C_max_ = 8.74 ± 2.68 ng/ml	
Oral	Jiaotai Pills extracts	Rat (plasma)	300 mg/kg Rhizoma Coptidis extracts and 4.7 mg/kg cinnamon oil (equivalent to 15.52 mg/kg dose of jatrorrhizine)	T_max_ = 5.25 ± 2.22 min	He et al. (2014a)
				T_1/2_ = 3.88 ± 1.46 h	
				AUC_0-t_ = 10.36 ± 4.28 ng h^−1^·mL^−1^	
				AUC_0-∞_ = 11.11 ± 4.63 ng h^−1^·mL^−1^	
				K_e_ = 0.20 ± 0.08 h^−1^	
				C_max_ = 1.04 ± 0.67 ng/ml	
		Insomnic rat (plasma)		T_max_ = 0.53 ± 0.30 min	
				T_1/2_ = 8.94 ± 15.99 h	
				AUC_0-t_ = 9.47 ± 2.25 ng h^−1^·mL^−1^	
				AUC_0-∞_ = 13.22 ± 4.69 ng h^−1^·mL^−1^	
				K_e_ = 0.33 ± 0.20 h^−1^	
				C_max_ = 8.64 ± 2.17 ng/ml	
i.v.	Jatrorrhizine	Rat (plasma)	0.1 mg/kg	T_1/2_ = 8.5 ± 2.6 h	Shi et al. (2012)
				AUC_0-t_ = 7.6 ± 2.9 μg h^−1^·L^−1^	
				AUC_0-∞_ = 9.6 ± 3.6 μg h^−1^·L^−1^	
				V_d_ = 188.9 ± 121.7 L/kg	
				CL = 11.6 ± 3.8 L/h/kg	
				MRT_0-t_ = 5.7 ± 2.3 h	
			0.3 mg/kg	T_1/2_ = 10.6 ± 5.4 h	
				AUC_0-t_ = 29.9 ± 13.1 μg h^−1^·L^−1^	
				AUC_0-∞_ = 32.1 ± 13.4 μg h^−1^·L^−1^	
				V_d_ = 149.9 ± 74.4 L/kg	
				CL = 10.6 ± 3.9 L/h/kg	
				MRT_0-t_ = 8.3 ± 4.2 h	
			3 mg/kg	T_1/2_ = 8.9 ± 2.2 h	
				AUC_0-t_ = 307.8 ± 85.9 μg h^−1^·L^−1^	
				AUC_0-∞_ = 308.9 ± 85.7 μg h^−1^·L^−1^	
				V_d_ = 137.0 ± 57.5 L/kg	
				CL = 10.3 ± 2.8 L/h/kg	
				MRT_0-t_ = 8.8 ± 1.4 h	
Oral	San-Huang decoction	Rabbit (plasma)	7.67 ml/kg (equivalent to 7.13 mg/kg dose of jatrorrhizine)	T_max_ = 0.50 ± 0 h	Liu et al. (2011)
				T_1/2_ = 18.12 ± 4.74 h	
				AUC_0-∞_ = 1,099.54 ± 292.67 h ng/ml	
				C_max_ = 71.30 ± 7.72 ng/ml	
Oral	Coptis–evodia powder (6:1, g/g)	Rat (plasma)	1.086 g/kg (equivalent to 14.4 mg/kg dose of jatrorrhizine)	T_max_ = 90 ± 0 min	Deng et al. (2008)
				T_1/2_ = 325.3 ± 8.0 min	
				AUC_0-∞_ = 43,576.9 ± 4,767.8 ng min/ml	
				C_max_ = 219.9 ± 12.8 ng/ml	
Oral	Coptis Root extract	Rat (plasma)	800 mg/kg	T_max_ = 0.67 ± 0.23 h	Zhao et al. (2018)
				T_1/2_ = 8.6 ± 2.61 h	
				AUC_0-t_ = 7.5 ± 0.87 ng h/mL	
				AUC_0-∞_ = 8.6 ± 0.80 ng h/mL	
				C_max_ = 3.12 ± 0.84 ng/ml	
	Shuanghua Baihe tables powder		3.13 g/kg	T_max_ = 4.2 ± 0.53 h	
				T_1/2_ = 11.1 ± 2.06 h	
				AUC_0-t_ = 7.7 ± 2.02 ng h/mL	
				AUC_0-∞_ = 11.8 ± 3.06 ng h/mL	
				C_max_ = 1.53 ± 0.20 ng/ml	
Oral	Coptidis Rhizoma extract	Rat (plasma)	0.0650 g/200 g	C_max_ = 33.35 ± 5.82 μg/L	Sun et al. (2018)
				AUC_0-tn_ = 96.58 ± 21.69 ug/L h	
				MRT_0-tn_ = 6.14 ± 0.30 h	
				VRT_0-tn_ = 15.45 ± 1.26 h^2^	
	JinQi Jiangtang tablets		0.4536 g/200 g	C_max_ = 11.35 ± 2.48 μg/L	
				AUC_0-tn_ = 279.70 ± 83.40 ug/L h	
				MRT_0-tn_ = 5.08 ± 0.42 h	
				VRT_0-tn_ = 24.55 ± 5.42 h^2^	
Oral	Coptidis Rhizoma powder	Rat (plasma)	1.08 g/kg	T_max_ = 0.75 ± 0.11 h	Yan et al. (2012)
				T_1/2_ = 7.1 ± 6.4 h	
				AUC_0-t_ = 123.1 ± 31.1 μg/L·h	
				AUC_0-∞_ = 128.9 ± 37.4 μg/L·h	
				MRT_0-t_ = 3.5 ± 0.8 h	
				MRT_0-∞_ = 4.9 ± 3.8 h	
				C_max_ = 82.09 ± 17.44 μg/L	
	Zoujinwan		Rhizoma coptidis powder 1.08 g/kg and Evodia rutaecarpa powder 0.18 g/kg	T_max_ = 1.50 ± 0.89 h	
				T_1/2_ = 8.0 ± 3.7 h	
				AUC_0-t_ = 107.9 ± 50.8 μg/L·h	
				AUC_0-∞_ = 113.8 ± 48.1 μg/L·h	
				MRT_0-t_ = 4.3 ± 0.9 h	
				MRT_0-∞_ = 5.9 ± 3.0 h	
				C_max_ = 39.63 ± 13.35 μg/L	

T_max_: the time of maximum plasma concentration; T_1/2_: the elimination half-life; AUC: area under the concentration-time curve; C_max_: maximum plasma concentration; K_e_: eliminate rate constant; V_d_: apparent volume of distribution; CL: clearance; MRT: mean residence time; VRT: the variance of residence time.

Cui et al. (2015) reported permeability and absorption of jatrorrhizine in rats after oral administration. An apparent permeability coefficient of jatrorrhizine (0.23–0.36 × 10^−6^ cm s^−1^) indicated limited ability to cross cell membranes. P-glycoprotein (P-gp) efflux had a significant effect on the absorption of this compound, which may explain its poor bioavailability. Intestinal perfusion experiments confirmed absorption into the rat jejunum (8.98 ± 2.43%) and ileum (7.54 ± 1.45%) (Cui et al., 2015). Jiaotai Pill extracts containing jatrorrhizine (15.52 mg/kg) was administered orally to rats and the pharmacokinetic parameters in rat brain were assessed using LC-MS/MS. The half-life of terminal elimination phase (T_1/2_), AUC _(0-t)_, AUC _(0-∞)_, and C_max_ in insomnic rats were increased compared with normal controls (Zhang et al., 2018). Thus, the absorption and bioavailability of jatrorrhizine may increase under pathological conditions. A study on plasma pharmacokinetics reported similar results (He W. et al., 2014). Rat plasma jatrorrhizine concentrations showed a biphasic decline, dose-independent clearance (C_L_) and T_1/2_, and dose dependent AUC _(0-∞)_ after intravenous administration (0.1 mg/kg to 3 mg/kg). Large distribution volumes (V_d_) indicated that jatrorrhizine might be distributed across tissues (Shi et al., 2012). Also, three peaks were observed in both individual and mean plasma-concentration curves of jatrorrhizine in rats after oral gavage with Coptis–Evodia powder (1.086 g/kg). This finding may be explained by distribution, re-absorption and enterohepatic circulation (Deng et al., 2008). In addition, absorption and elimination of jatrorrhizine are also influenced by compounds and other herbs coexisting with it, such as Astragali Radix, Lonicerae Japonicae Flos and Fructus Evodiae (Yan et al., 2012; Sun et al., 2018; Zhao et al., 2018). It is required to explore and confirm the compound-compound and compound-herb interaction mechanism in the further research.

Biotransformation of jatrorrhizine was similar among liver microsomes from rats, rhesus monkeys and humans. C_20_H_20_O_7_N was the major metabolite in these species (Li et al., 2015). Seventeen metabolites in rat urine, thirteen metabolites in rat faeces (including eight phase I metabolites and five phase II metabolites) and eleven metabolites in rat plasma (including six phase I metabolites and five phase II metabolites) were detected after oral administration of jatrorrhizine (34 mg/kg) to healthy rats. Further, seventeen and nine metabolites were identified after incubating jatrorrhizine with rat intestinal flora and liver microsomes, respectively (Zhang et al., 2008). Also, jatrorrhizine metabolism exhibited high consistency between human and zebrafish (Li et al., 2015). Additionally, seven phase I metabolites of jatrorrhizine after demethylation, dehydrogenation and dihydroxylation, and eleven phase II metabolites including glucuronide and methyl conjugates were detected in rat urine (Han et al., 2006). Cytochrome P450 and UDP-glucuronosyltransferase enzymes are responsible for the metabolism of jatrorrhizine in human liver microsomes, including CYP1A2 and multiple UGT1A isoforms (UGT1A1, UGT1A3, UGT1A7, UGT1A8, UGT1A9 and UGT1A10) (Zhou et al., 2013). CYP3A1/2 and CYP2D2 were also involved in demethylation of jatrorrhizine and UGT1A1 and UGT 1A3 were associated with glucuronidation in rat liver microsomes (Shi et al., 2012).

## Toxicity of Jatrorrhizine

Toxicity and safety are critical for the assessment of clinical applications of natural products (Li et al., 2021). Jatrorrhizine is a bioactive metabolite in some commonly used medicinal plants and has been used for centuries in traditional medicine. However, analyses of the composition of traditional medicines and modern pharmacology research indicate that some natural products may produce adverse effects or even overt toxicity under certain conditions despite their beneficial pharmacological properties. Jatrorrhizine exhibited anti-cancer activity on SW480 cells (human colon cancer) and HepG2 cells (hepatocellular carcinoma) as discussed above. However, it also showed cytotoxicity against these cancer cells the at the concentrations of 200 μg/ml and 100 μM, respectively (Chen et al., 2012; Singh et al., 2016). Slight cytotoxicity was also reported in normal MCF10A normal breast eptithelial cells *in vitro* (100 μM) (Sun et al., 2019). However, jatrorrhizine did not cause cytotoxicity to MCF-7 cells at the concentration below 10 µM (Lo et al., 2017). Additionally, Sun et al. (2019) reported that gross necropsy did not show signs of toxicity in the jatrorrhizine-treated (2.5 and 5 mg/kg) 4T1tumour-bearing mice (Sun et al., 2019). Also, there was no significant changes in body weight and serum ALT and AST levels in jatrorrhizine (25 and 100 mg/kg)-treated Ti particle-induced mice, as compared with the untreated and control groups (Li et al., 2018). The alkaloid was also non-cytotoxic to rheumatoid arthritis-derived fibroblast-like synoviocyte MH7A cells, and no damage to liver function was observed in CIA rats at administered doses of 20 and 50 mg/kg (Qiu et al., 2018).

An acute LD_50_ value for jatrorrhizine was about 5,500 mg/kg in Kunming mice, and significantly higher than that of the related alkaloid, berberine (763 mg/kg). No influences on body weight and organ weight were observed in rats in a sub-chronic study, and no abnormalities in urinalysis and haematological parameters, gross necropsy or histology were reported after administration of jatrorrhizine (70.05 mg/kg day) over 90 days (Wu et al., 2014). Few indications of adverse effects of jatrorrhizine are available, but we should explore mechanisms for any toxicity observed in particular clinical circumstances.

## Conclusion and Future Perspectives

**FIGURE 5:**
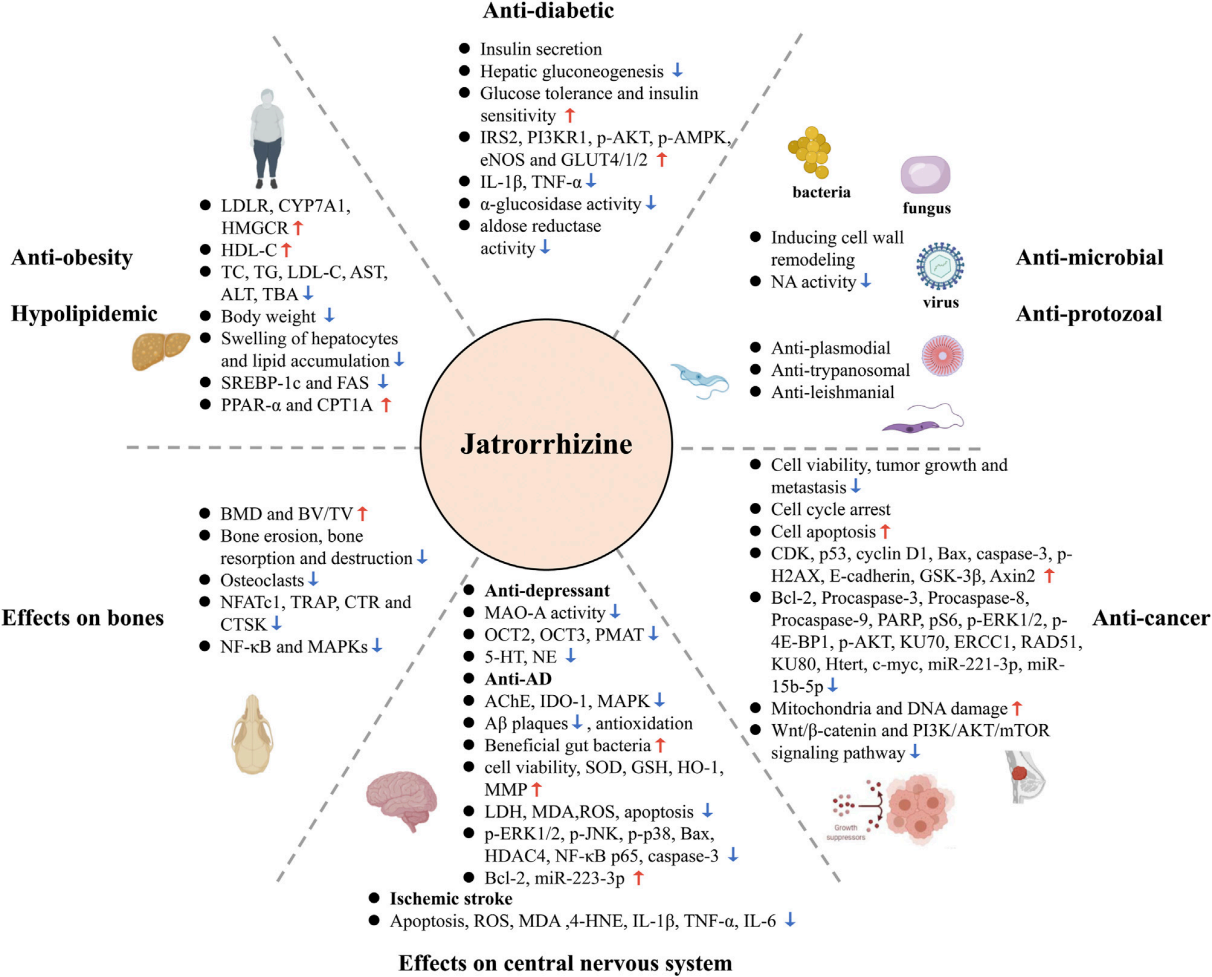
Summary of the therapeutic potential of jatrorrhizine through multiple pathways and multiple targets.

Jatrorrhizine is encountered in a variety of traditional medicinal plants. This bioactive metabolite possesses numerous pharmacological properties *via* modulation of multiple signaling pathways and targets (Figure 5), such as AR, NA, MAO-A, OCT, PMAT, AChE, IDO-1, TNIK, and Wnt/β-catenin, MAPK, PI3K/AKT/mTOR, NF-κB, PPAR, and insulin signaling pathway. This review summarizes molecular mechanisms for possible use in the treatment of diseases, discusses pharmacokinetic parameters and evaluates toxicity and safety. However, some issues still need clarification in the future studies.

### Curative Mechanisms of Jatrorrhizine and Clinical Validity Confirmation

We recognise a potential for jatrorrhizine to be a therapeutic ingredient in medications, reflecting its impacts on multiple pathways and targets. However, its specific mechanisms of action against various diseases are not fully understood. Clarification the molecular targets of jatrorrhizine is conducive to a more scientific understanding and development of this natural metabolite. Currently, this natural metabolite is rarely alone used clinically to treat specific diseases even though it is the main active substance in some medicinal materials and extracts and has a role in the treatment of diabetes, gastrointestinal diseases, and Alzheimer’s disease in traditional medicine. Proper positive controls are necessary for future work to ensure the reproducibility and data quality for comparisons of therapeutic effects on various diseases and different disease stages. Sufficient evidence is available to support the detailed assessment of curative mechanisms of jatrorrhizine, structure-activity studies and clinical trials in humans. Such work should be approached systematically to fully explore the clinical utility of the alkaloid.

Jatrorrhizine is widely reported to exhibit pro-apoptotic effects in multiple cancer cells. However, it displays a protective role in AD and ischaemic stroke *via* reducing apoptosis. Mechanisms underlying these disparate effects require elucidation to understand actions on apoptosis in neuroprotection and cancer therapy, and subsequently improve targeting and specificity of jatrorrhizine through structural modification and dosage form optimization (e.g., as nanoparticles and liposome).

### Application Prospect of Jatrorrhizine in the Treatment of Metabolic Disorders

Long-term metabolic disorders and hyperglycemia remain a challenge in medical practice. These conditions cause a series of complications, such as cardiovascular disease, retinopathy, neuropathy and nephropathy. Current therapeutics for metabolic diseases require a multi-drug regimen. However, major problems of this therapeutic method are poor patient compliance, side effects and drug-drug interactions (Lillich et al., 2021). Multi-target ligands and drugs have been proposed as promising approaches to developing therapies for complex diseases (González-Álvarez et al., 2021). Jatrorrhizine is a multi-purpose natural metabolite that affects multiple targets. The alkaloid effectively modulates glucose and lipid metabolism and exhibits anti-inflammatory, anti-oxidant and anti-cancer effects. It is also a safe and controllable natural product. Therefore, the use of jatrorrhizine, alone or as a supplement to other nutraceuticals, is a potential strategy to address multiple pathways and targets to delay metabolic disorders and affect the long-term treatment of related complications.

### Comprehensive Investigations of Toxicity Mechanisms

We found that jatrorrhizine exerts cytotoxic effects under specific circumstances *in vitro*. High dose and long-term administration may lead to cytotoxicity in a few cancer cell lines, such as colon cancer and hepatocellular carcinoma cells and normal breast epithelial cells. However, there was no studies that report target-organ toxicity of jatrorrhizine in different disease models. The existing *in vivo* studies indicated that jatrorrhizine is non-cytotoxic and has no influence on liver function or other tissues. Therefore, further comprehensive investigation for mechanisms of toxicity is needed and further exploration of whether jatrorrhizine has target organ toxicity under special circumstances *in vivo* is of significance. Such studies will serve as a basis to further evaluate the safety of jatrorrhizine in the treatment of different diseases and for chronic administration.

### Interaction Mechanism of Jatrorrhizine With Other Compounds and Development of Derivatives

Jatrorrhizine displays low permeability and poor bioavailability. Interestingly, jatrorrhizine may interact with other constituents and thereby alter its absorption and elimination. Additionally, different salt forms of quaternary ammonium compounds show varying physicochemical properties. Thus, salts of jatrorrhizine might exhibit different pharmacokinetic properties *in vivo* (Neef et al., 1984; Cui et al., 2019). Berberine, an alkaloid similar in chemical structure to jatrorrhizine, displays better bioavailability of its organic acid salts (fumarate, malate, succinate and citrate) than inorganic acid salts (hydrochloride) (Cui et al., 2019). The hydrochloride salt is commonly used in clinical practice and pharmacological research. However, almost no reports on the pharmacokinetics of other salts and comparative studies of different salt forms are available for jatrorrhizine. Therefore, the investigation of interactions between jatrorrhizine and other compounds and the effects of different salt forms on pharmacokinetics is crucial. The improvement of the bioavailability of jatrorrhizine and development of jatrorrhizine derivatives with high bioavailability and low toxicity also needs to be explored.

## Abbreviations

3OHase, tyrosine/tyramine 3-hydroxylase; 4-HNE, 4-hydroxynonenal; 4HPPDC, 4-hydroxyphenylpuruvate decarboxylase; 4′OMT, 3′-hydroxy-N-methyl-(S)-coclaurine 4′-O-methyltransferase; 53BP1, p53-binding protein 1; 5-HT, serotonin; 6OMT, (S)-norcoclaurine 6-O-methyltransferase; Aβ, amyloid β; AChE, acetylcholinesterase; Apaf-1, apoptotic protease activating factor 1; ALT, alanine aminotransferase; AR, aldose reductase; AST, aspartate transaminase; Bax, BCL-2-associated X protein; BBE, berberine bridge enzyme; Bcl-2, B cell lymphoma 2; BMD, bone mineral density; BV/TV, bone volume/tissue volume; CDK, cyclin-dependent kinase; CNMT, (S)-coclaurine N-methyltransferase; CPT1A, carnitine palmitoyltransferase 1A; CODM, codeine-O-demethylase; CTR, calcitonin receptor; CTSK, cathepsin K; CYP7A1, cholesterol 7α-hydroxylase; eNOS, endothelial nitric oxide synthase; EMT, epithelial-mesenchymal transition; ERCC1, excision repair cross-complementation group 1; FAS, fatty acid synthase; GLUT4/1/2, glucose transporter 4/1/2; GSH, glutathione; GSK-3β, glycogen synthase kinase-3β; HDAC4, histone deacetylase 4; HDL-C, high-density lipoprotein cholesterol; HMGCR, 3-hydroxy-3-methyl glutaryl coenzyme A reductase; HO-1, heme oxygenase-1; hTERT, human telomerase reverse transcriptase; IDO-1, indoleamine 2, 3-dioxygenase 1; IL-1β, interleukin-1β; IRS2, insulin receptor substrate 2; LDH, lactate dehydrogenase; LDL-C, low-density lipoprotein cholesterol; LDLR, low density lipoprotein receptor; MAO, monoamine oxidase; MAPK, mitogen-activated protein kinases; MDA, lipid peroxidation; MIC, minimum inhibitory concentration; MMP (∆Ψm), mitochondrial membrane potential; NA, neuraminidase; NCS, (S)-norcoclaurine synthase; NE, norepinephrine; NFATc1, T-cells cytoplasmic 1; NF-κB, nuclear factor kappa-B; NMCH, (S)-N-methylcoclaurine-3′-hydroxylase; OCT, organic cation transporter; OMT, O-methyltransferase; p-4E-BP1, phosphorylated 4E-binding protein 1; PARP, ADP-ribose polymerase; p-AKT, phosphorylated protein kinase B; p-AMPK, phospho-AMP-activated protein kinase; p-ERK1/2, phosphorylated extracellular signal-regulated kinases 1/2; pH2AX, phosphorylated H2A histone X; PI3KR1, phosphoinositide-3-kinase regulatory subunit 1; p-JNK, phosphorylated c-Jun N-terminal kinases; PMAT, plasma membrane monoamine transporter; PPAR, peroxisome proliferator activated receptor; p-p38, phosphorylated p38; ROS, oxygen species; SOMT, (S)-scoulerine 9-O-methyltransferase; SREBP-1c, sterol regulatory element binding transcription factor 1c; STOX, (S)-tetrahydroprotoberberine oxidase; T2DM, type 2 diabetes mellitus; TBA, total bile acids; TC, total cholesterol; TRF1, TTAGGG repeat binding factor 1; TG, total triglyceride; TNF-α, tumor necrosis factor α TRAP, tartrate-resistant acid phosphatase; TYDC, tyrosine decarboxylase; TyrAT, tyrosine aminotransferase.
